# Suffering and mental health among older people living in nursing homes—a mixed-methods study

**DOI:** 10.7717/peerj.1120

**Published:** 2015-07-30

**Authors:** Jorunn Drageset, Elin Dysvik, Birgitte Espehaug, Gerd Karin Natvig, Bodil Furnes

**Affiliations:** 1 Faculty of Health and Social Sciences, Bergen University College, Norway; 2 Department of Global Public Health and Primary Care, University of Bergen, Norway; 3 Department of Health Studies, Faculty of Social Sciences, University of Stavanger, Norway

**Keywords:** Suffering, Mental health, Mix-methods, Nursing homes

## Abstract

**Background.** Knowledge about mixed-methods perspectives that examine anxiety, depression, social support, mental health and the phenomenon of suffering among cognitively intact NH residents is scarce. We aimed to explore suffering and mental health among cognitively intact NH residents.

**Methods.** This study used a mixed-methods design to explore different aspects of the same phenomena of interest to gain a more comprehensive understanding. The qualitative core component comprised a qualitative interview from 18 nursing home residents (≥65 years) about experiences related to pain, grief and loss. The supplementary component comprised interview from the same respondents using the SF-36 Health Survey subscales, the Hospital Anxiety and Depression Scale and the Social Provisions Scale.

**Results.** The individual descriptions reveal suffering caused by painful experiences during life. The quantitative results indicated that symptoms of anxiety and depression were related to mental health and symptoms of anxiety were related to bodily pain and emotional role limitations. Attachment and social integration were associated with vitality and social functioning.

**Discussion.** To improve the situation, more attention should be paid to the residents’ suffering related to anxiety, depression and psychosocial relations.

## Introduction

Nursing home (NH) residents without cognitive impairment comprise a minority of NH residents and often have somatic and/or other mental health conditions (Nygaard, Naik & Ruths, 2000; Linton & Lach, 2007; Selbaek et al., 2007). NH residents may also experience stressful events such as relational losses, loss of home and loss of spouse, relatives and friends. Such conditions may cause them to experience suffering (Morse, 2001; Cassel, 2004; Ferrell & Coyle, 2008) and affect both mental and physical health (Travis et al., 2004; Garcia et al., 2005). Morse (2011) describes suffering as a basic emotional experience that follows illnesses that threaten one’s physical or psychosocial integration. Thus, suffering refers to human existence as a whole, whereas experiencing suffering can mean being unable to keep oneself together as a whole entity. Alleviating suffering implies lessening people’s sense of vulnerability (Rehnsfeldt & Eriksson, 2004). Studies from Canada (Daneault et al., 2004) and Finland (Kuuppelomaki & Lauri, 1998) found that people report suffering in three dimensions; physical, mental and social well-being. Physical suffering includes pain, mental suffering is mostly expressed as depression and social suffering is often caused by disease that results in isolation and withdrawal from social contact (Kuuppelomaki & Lauri, 1998).

Depression is common among older people living in NHs (Smalbrugge et al., 2005a; Smalbrugge et al., 2006a; Drageset, Eide & Ranhoff, 2011; Drageset, Eide & Ranhoff, 2013); the prevalence is 34–41%. Depression is often experienced together with anxiety, where anxiety tends to follow traumatic events and loss situations (Bland, 2012), such as loss of significant others but also functional abilities and loss caused by disease.

Only a few studies of the associations between the experience of anxiety and depression and quality of life among older cognitively intact people in NHs have been published (Smalbrugge et al., 2006b; Rodriguez-Blazquez et al., 2012). In Spain, Rodriguez-Blazquez et al. (2012) found that depression, health status and the interaction of age and institutionalization were related to well-being. In a study in the Netherlands among NH residents, Smalbrugge et al. (2006b) studied the impact of depression and anxiety on well-being and found that depression and/or anxiety were associated with significantly worse psychosocial well-being.

Another source of suffering may be social isolation, resulting from difficulty in maintaining social contact because of disabilities and loss of spouse and friends (Drageset, Kirkevold & Espehaug, 2010). For some NH residents, it means that they no longer have anyone close in their social network. The experience of suffering may be a reaction to the lack of someone close (Morse, 2011).

Social support may alleviate the experience of suffering and, in turn, may affect mental and physical health. Social support involves qualitative aspects of human relationships, such as the content and the availability of relationships with significant others (Sarason, Sarason & Pierce, 1990). Social support is conceptualized in the literature from three dimensions (affect, affirmation and aid (Kahn, 1979)) to five dimensions (emotional support, esteem support, material support, instrumental support and active support (Cobb, 1979; Cohen, Lynn & Gottlieb, 2000)). Most authors include dimensions related to emotional support, confirmation support and practical support. Few relevant studies of social support and quality of life among mental intact NH residents have been conducted. In a quantitative study, Tseng & Wang (2001) found that social support from nursing aides and the frequency of interaction with family significantly predict the quality of life of NH residents. In a qualitative NH study in Norway, Bergland & Kirkevold (2005) found that residents reported positive peer relationships, and positive relationships with family were essential for thriving. A qualitative study in Canada (Welsh, Moore & Getzlaf, 2012) explored meaning in life for NH residents. Connectedness and engaging in “normal” activities emerged as enhancing meaning in life for the residents. A systematic qualitative review of NH life in relation to residents’ quality of life reported connectedness with others as being essential for residents’ quality of life (Bradshaw, Playford & Riazi, 2012).

We have recently found that NH residents reported symptoms of anxiety and depression (Drageset, Eide & Ranhoff, 2013), and some dimensions of social support (attachment, nurturance and reassurance of worth) have been shown to be important for health-related quality of life (Drageset et al., 2009a). Based on our recent findings and the present literature review, one may question that cognitively intact NH residents who report symptoms of anxiety and depression and a lack of social contact are particularly prone to reduced health-related quality of life (HRQOL) and the increased experience of suffering.

Some studies have investigated the association between anxiety, depression, social support and HRQOL among cognitively intact NH residents (Kuuppelomaki & Lauri, 1998; Tseng & Wang, 2001; Bergland & Kirkevold, 2005; Drageset et al., 2009a; Bradshaw, Playford & Riazi, 2012; Welsh, Moore & Getzlaf, 2012; Drageset, Eide & Ranhoff, 2013), but we found no relevant studies about mixed-methods perspectives that examine this association and the phenomenon of suffering. Better care and treatment for older people with no cognitive impairment living in NHs requires focusing on well-being at a holistic level. This means including emotional well-being and the availability of meaningful social relationships. Meeting this challenge requires extending the perspectives beyond what one specific research approach can give.

## Aim

The aim of this study was to investigate suffering and mental health among cognitively intact NH residents more broadly by using a mixed-methods design. The specific research questions were as follows.

• How do older people living in NHs experience their life situation?
• What is the association between sociodemographic and illness variables and anxiety, depression and health-related quality of life for NH residents?
• How can the quantitative findings extend findings from qualitative analysis for NH residents?

## Methods

### Design and population

Since we wanted to explore different aspects of the same phenomena, we used a qualitative mixed-methods design with concurrently components: qualitative and quantitative (Morse & Niehaus, 2009). The theoretical drive or the inductive direction of a research project guides the qualitative methodological core (Morse et al., 2006) and allows researchers to explore experiences qualitatively and to build a theoretical model of suffering during anxiety, depression and social support subdimensions and health-related quality of life. These methods differ from other approaches because the qualitative core component is guiding the study, which may stand alone, and the supplementary quantitative component is used to expand certain details of the findings to indicate the validity of the core findings.

The qualitative core component in the study comprised a qualitative interview about life experiences related to pain, grief and loss and psychosocial topics from 18 respondents. The supplementary component in the study comprised the same 18 respondents, in which we interviewed the participants face to face using the SF-36 Health Survey subdimensions bodily pain, vitality, social functioning, role–emotional and mental health; the Hospital Anxiety and Depression Scale (HADS); and the Social Provisions Scale (SPS). The supplementary component (findings from the quantitative analysis) enabled us to explore significant relationships and may inform the qualitative findings.

**Figure 1:**
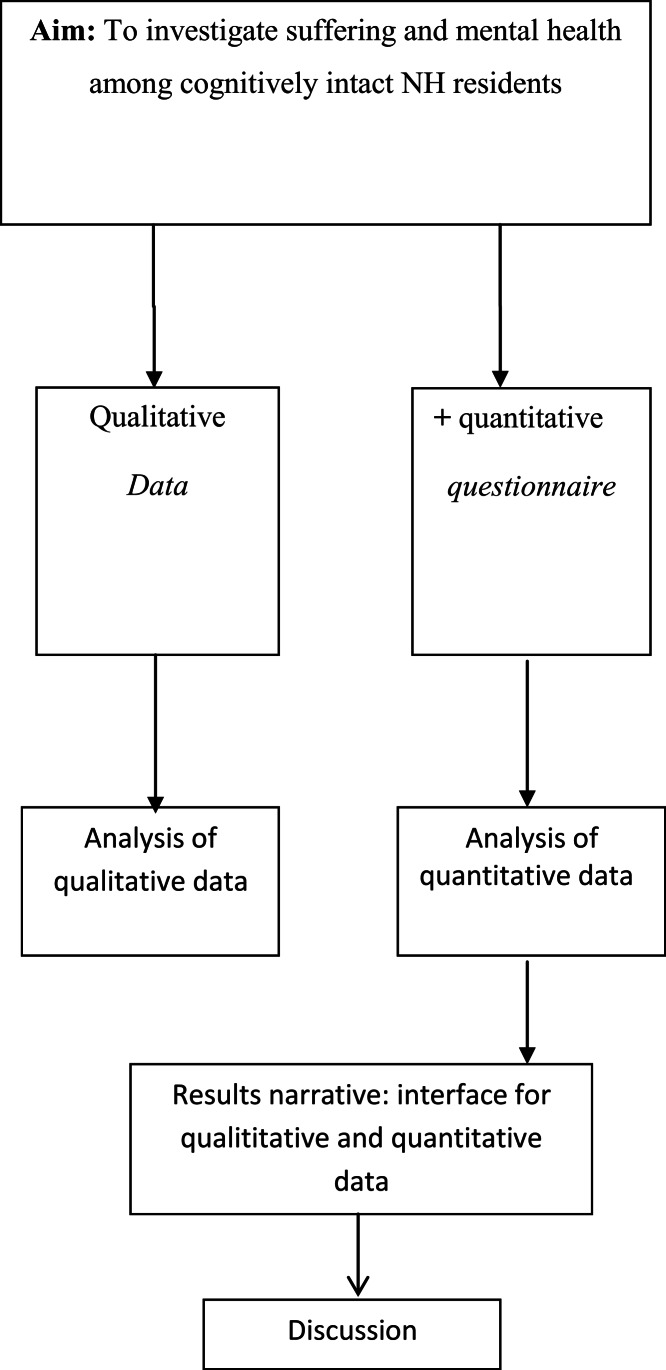
Schematic overview. The left pathway illustrates the core component of the project (qualitative data). The right pathway illustrates the supplemental components of the project (qualitative data). The point of interface is the position at which the core and supplemental components meet. The “results narrative” refers to the write-up of the core-component findings with the addition of the results of the supplemental components.

This study used a simultaneous design (Fig. 1). Once we analyzed the qualitative core component and completed the supplementary components, we first described the findings on the core component. We then integrated the final descriptions from the quantitative components, and these constitute a results narrative on which the discussion is based (Fig. 1).

This study is part of a study conducted in 2004–2005 (Drageset et al., 2009b), with follow-up until 2011. At the end of follow-up, 19 NH residents were still alive, and 18 were included based on the inclusion criteria: aged 65 years and older, cognitively intact, capable of carrying out a conversation and residing in the NH for at least 6 months. Cognitively intact was defined as having a Clinical Dementia Rating (CDR) ≤0.5 (Hughes et al., 1982). CDR was developed as a staging instrument for dementia and is scored as no (0), questionable (0.5), mild (1), moderate (2) and severe (3) dementia, and the overall level of dementia is derived by using a standard algorithm (Morris, 1993). Trained nurses who had observed the residents for at least 4 weeks assessed CDR and were instructed to base their CDR scoring on mental functioning and not to include physical frailty. The CDR has shown high interrater reliability for physicians and other health professionals (McCulla et al., 1989). Exclusion criteria were: lived shorter than 6 months in an NH, CDR score >0.5 and those who had their general health status assessed by a doctor or nurse, who indicated that the residents could not converse with the researcher. A primary care nurse invited them to participate.

### Ethical approval

The project involved a sample collected during 2010–2011. We obtained informed consent. The Western Norway Regional Committee for Medical and Health Research Ethics and the Norwegian Social Science Data Services approved the study (REK.Vest nr. 62.03/2009/1550).

### Data collection

#### Qualitative data

We collected data through individual interviews. We developed a semistructured interview guide based on our previous research findings. We asked informants about mental health and physical health (including their experience of depression, anxiety, loss and pain). We performed interviews in the residents’ room, and they lasted 45–90 min. The interviewer (first author) conducted conversations in which participants were encouraged to describe their experience regarding the questions in the interview guide. The respondents’ answers to the questions also led to spontaneous follow-up questions from the interviewer. The 18 interviews were taped and transcribed verbatim. In all, this resulted in detailed descriptions.

#### Quantitative data

We obtained the quantitative data through face-to-face data collection. The 18 interviews took place in the respondent’s room or at another appropriate location in the NH. The principal investigator (JD) performed the quantitative interviews: read the questions to the participants, circled the indicated answer and recorded the demographic information. This was necessary, since many of the residents have problems holding a pen and have reduced vision.

#### Assessment instruments

We collected sociodemographic variables such as age, sex, marital status, education level and comorbidity from patient records. We scored comorbidity using the Functional Comorbidity Index, a clinically based measure (Groll et al., 2005). This index includes 18 diagnoses scored “yes = 1” and “no = 0.” A maximum score of 18 indicates the highest number of comorbid illnesses.

We measured health-related quality of life using the SF-36 (Ware & Sherbourne, 1992; Ware, 2005). The SF-36 comprises 36 questions along eight dimensions: physical functioning (10 items), general health (five items), mental health (five items), bodily pain (two items), role limitation related to physical problems (four items), role limitation related to emotional problems (three items), social functioning (two items) and vitality (four items). An additional item, reported health transition, notes changes in general health during the past year. The response scores for each dimension are added, and the total is converted to a score on a scale from 0 (poor health) to 100 (optimal health) (Ware, 2005). In this study, we used the subdimensions bodily pain, vitality, social functioning, role–emotional and mental health. The SF-36 has been used in numerous studies in geriatrics and appears to be reliable: Cronbach’s alpha: 0.68–0.94 (Lyons et al., 1994; Berlowitz et al., 1995; Pit et al., 1996). It has also demonstrated good construct validity (Lyons et al., 1994) and convergent validity (Andresen et al., 1999).

We used HADS (Zigmond & Snaith, 1983) to assess depression and anxiety symptoms. HADS is well validated (Bjelland et al., 2002). HADS has seven items for anxiety (HADS-A) and seven for depression (HADS-D). Each item is scored on a four-point scale from 0 (not present) to 3 (considerable). The maximum score is 21 on each subscale, and a higher score indicates a higher symptom load (Zigmond & Snaith, 1983). A score of 8–10 indicates possible cases of anxiety or depression, and a score ≥11 indicates cases of anxiety or depression that require further investigation and possibly treatment (Zigmond & Snaith, 1983). HADS has been translated into Norwegian and has been found to be reliable and valid for older people (Stordal et al., 2001; Stordal et al., 2003) and reliable and valid among NH residents (Haugan & Drageset, 2014).

We assessed social support using the revised SPS (Cutrona & Russell, 1987). The scale contains 24 items, four for each of the six social provisions worked out in detail by Weiss (1974). Russell et al. (1984) simplified the response format to a four-point rating scale: “strongly disagree,” “disagree,” “agree” and “strongly agree.” According to Cutrona & Russell (1987), Andersson & Stevens (1993) and Mancini & Blieszner (1992), four of the original six subscales were selected: “attachment” (emotional closeness from which one drives a sense of security); “social integration” (relationships in which the person shares concerns and common interests); “opportunities for nurturance” (being responsible for the care of others); and “reassurance of worth” (a sense of competence and esteem). High scores indicate high social provision. The SPS, with four subdimensions “attachment,” “social integration,” “nurturance” and “reassurance of worth,” has been used in studies of older people living in the community (Andersson & Stevens, 1993; Bondevik & Skogstad, 1996; Saevareid et al., 2010) and among NH residents (Bondevik & Skogstad, 1998; Drageset, 2002; Drageset et al., 2009b).

### Data analysis

#### Qualitative data

We analyzed the interview data by using elements of qualitative content analysis (Graneheim & Lundman, 2004; Kvale & Brinkmann, 2009). The analytical process occurred in six stages (see Table 3 for details): (1) transcribing the interview; (2) open independent reading of all material to gain an overall impression of the text; (3) identifying meaning units; (4) categorization; (5) abstracting two subthemes and a theme (Table 3); and (6) reflection and discussion.

**Table 1:** Characteristics of the respondents.

	*n*	%
	18	100
**Sex**		
Male	7	38.9
Female	11	61.1
**Age (years)**		
65–74	3	16.7
75–84	7	38.9
85–94	8	44.4
≥95	0	0
**Marital status**		
Widowed	11	61.1
Married or cohabiting	4	22.2
Unmarried	3	16.7
**Education**		
Lowest: primary school	8	44.4
Middle: <3 years after primary school	4	33.3
Highest: ≥3 years after primary school	4	22.2
**Illnesses** ^a^		
Yes	17	94.1
No	1	5.9

**Notes.**

a Functional Comorbidity Index includes 18 diagnoses scored “yes = 1” and “no = 0.” A maximum score of 18 indicates the highest number of comorbid illnesses.

**Table 2:** Means and standard deviation (SD) for each of five subscales of SF-36 according to sociodemographic and comorbid illnesses, and correlation coefficient for anxiety, depression and social support dimensions (*n* = 18).

	Bodily pain	Vitality	Social functioning	Role limitations, emotional	Mental health
**All**	64.1 (27.4)	43.9 (12.7)	81.2 (17.3)	72.2 (36.6)	68.4 (13.1)
**Sex** ^a^					
Women	61.7 (28.2)	46.8 (11.9)	78.4 (17.8)	66.7 (42.2))	66.2 (13–2)
Men	61.7 (28.2)	40.0 (13.4)	85.7 (16.8)	80.9 (26.2)	72.0 (13.1)
***P*** ^*^	0.791	0.211	0.425	0.659	0.425
**Age (years)** ^b^	−0.14	0.125	0.10	0.08	0.04
***P*** ^*^	(0.572)	(0.684)	(0.684)	(0.745)	(0.883)
**Marital status** ^c^					
Widowed	69.5 (29.4)	45.0 (13.6)	83.0 (16.7)	69.7 (40.7)	68.0 (13.6)
Married	61.2 (31.1)	47.5 (8.7)	81.2 (21.7)	75.0 (38.5)	70.0 (16.5)
Unmarried	48.3 (6.4)	35.0 (13.2)	75.0 (21.7)	77.8 (38.5)	68.0 (10.6)
***P*** ^*^	0.71	0.41	0.83	0.94	0.93
**Anxiety** ^b^	−0.56	−0.23	−0.34	−0.58	−0.86
***P*** ^*^	(**0.018)**	(0.385)	(0.178)	**(0.016)**	(**<0.001**)
**Depression** ^b^	−0.05	−0.23	−0.26	−0.16	−0.62
***P*** ^*^	(0.845)	(0.157)	(0.309)	(0.535)	(**0.007)**
**Attachment** ^b^	−0.145	0.562	0.257	−0.111	0.214
***P*** ^*^	(0.566)	**(0.015)**	(0.304)	(0.661)	(0.395)
**Social integration** ^b^	0.391	0.014	0.536	−0.108	−0.146
***P*** ^*^	(0.109)	(0.957)	**(0.022)**	(0.670)	(0.564)
**Reassurance of worth** ^b^	−0.407	0.334	0.258	−0.243	0.005
***P*** ^*^	(0.094)	(0.175)	(0.301)	(0.331)	(0.983)
**Nurturance** ^b^	0.430	0.035	0.030	−0.486	−0.125
***P*** ^*^	(0.075)	(0.889)	(0.907)	**(0.041**)	(0.622)
**Grolls index** ^b^ ^,^ ^d^	−0.412	−0.48	−0.15	−0.40	−0.44
***P*** ^*^	(0.101)	(0.050)	(0.555)	(0.108)	(0.078)

**Notes.**

a Mann–Whitney U test.

b Spearman correlation coefficient.

c Kruskal–Wallis test.

d Functional comorbidity index. A maximum score of 18 indicates the highest number of comorbid illnesses.

* bold, statistical significance at 0.05.

**Table 3:** Stages of the qualitative analytical process.

(1) Transcription	Data were transcribed and organized according to the interview guide
(2) Open reading	Two co-authors carefully and independently read and discussed the interview text to obtain an overall impression of the participants’ experiences
(3) Identifying meaning units	Patterns in the data were identified by dividing the text into meaning units
(4) Categories	Important nuances were discovered by searching for common and distinctive features as well as variation and agreement about suitable categories
(5) Forming themes	Two subthemes were formulated Thereafter, analytical reflection and abstraction were performed by searching for an overall theme
(6) Reflection and discussion	Dialogue was searched for relevant theory to illuminate and deepen understanding of the findings

#### Quantitative data

We used descriptive statistics for the demographic variables and the comorbidity variables. We applied nonparametric test statistics to test for distributional differences in SF-36 subscales among groups defined by sex (Mann–Whitney *U*-test) and marital status (Kruskal–Wallis test). We calculated Spearman correlation coefficients to study associations between comorbidity, age, anxiety, depression, social support dimensions and SF-36 subdimensions: bodily pain, social functioning, role–emotional, vitality and mental health.

## Results

### Respondents’ characteristics

Table 1 presents descriptive statistics for the demographic variables and the comorbidity variables. Of the 19 NH residents, 18 (95%) met the inclusion criteria; 1 (5%) declined to participate.

Of the 18 respondents, 11 (62%) were women. The mean age was 84.8 years (SD 7.6). The mean number of comorbid illnesses was 1.9 (median 2.0, SD 1.3, range 0–5).

### Qualitative data

During the interviews, the informants often strived to express their feelings verbally. Nevertheless, the qualitative data reveal many previous and current life experiences related to psychosocial aspects and suffering. Among these, the informants described several loss and traumatic experiences related to death (of parents, siblings, relatives and friends), war, starving and isolation. During the conversation, the individuals often return to stories from childhood and upbringing. According to the qualitative analysis, one main theme was lifelong suffering as a complex psychosocial entity, with two subthemes: “pain from experience in early life” and “painful experience in recent life” (Table 4).

**Table 4:** The qualitative content analysis.

Categories	Subtheme	Theme
**Earlier life experiences**	Painful experiences in earlier life	Suffering as a lifelong complex psychosocial entity
Loss by death		
Instability		
Lack of hope		
Mental strain		
Traumatic events		
**Present life experiences**	Painful experiences in recent life	
Loss by death		
Loss of health		
Lack of social relationships		
Lack of courage to live		
Lack of hope		

Such early-life experiences are often related to loss by death, instability and lack of hope in their upbringing. Several descriptions reflect this psychosocial complexity, and representative quotations are presented here to give the participants a voice:

• “I have a lifelong grief caused by traumatic experiences from the war.” (P)
• “I feel grief caused by difficult experiences during my childhood.” (Q)
• “Unstable upbringing and hopelessness have been painful.” (F)

The interviewees strongly emphasized earlier painful life experiences. Loss and grief seem to be especially attached to these.

Current life experiences are also related to loss by death, and lack of social relations, lack of courage to live and lack of hope are prominent. Many descriptions revealed resignation and hopelessness:

• “I just sit here.” (O)
• “I am in despair and lonely, but that’s life for us old people, and I cannot do anything about it.” (F)
• “I feel like a prisoner and several times I wish that I did not exist. I have had enough!” “I live in a cemetery.” (Q)
• “I am crying all day long.” (A)
• “I lie in bed, feel totally isolated, and cannot take care of myself.” (F)
• “Noise from other people all the time is quite stressful.” (D)

The qualitative data reveal clear patterns and similarities in the descriptions. In their present life situation, loneliness, despair and depressive thoughts are prominent, and they express several attempts to endure their situation.

• “If you are unable to come into contact with others, you will feel the loneliness strongly and you also feel invisible.” (P)

The current life experiences also contained several contradictory descriptions:

• “[Life] is very sad, it is terrible, but I have nothing to complain about.” (J)
• “…, I just have to accept.” (H)

The individual stories consistently described existence as a state the informants have to endure and adapt to. In this process, several expressed that good relationships (staff and relatives) and mobility are very important. The relationships with health care providers are especially important.

• “I do not always trust the health care workers, but they are okay.” (F)

Or more explicitly stated: “We need health care workers who care for us.”

### Quantitative data

In general, residents scored highest on role–emotional and social functioning and lowest on vitality (Table 2). Women reported lower, but not statistically significantly lower, scores than men on all subdimensions except for vitality and bodily pain.

The health-related quality of life subdimensions bodily pain (*P* = 0.02), role–emotional (*P* = 0.02) and mental health (*P* < 0.001) were negatively associated with anxiety. Increasing depression scores were negatively associated with mental health (*P* = 0.007). Attachment was positively associated with vitality (*P* = 0.02) and social integration with social functioning (*P* = 0.02). Nurturance was negatively associated with role–emotional (*P* = 0.04).

Cronbach’s alpha for the SF-36 subscales ranged from 0.60 to 0.85, with role–emotional showing the highest values and vitality the lowest.

## Results narrative

The results narrative summarizes the core component findings, with the supplementary component adding certain information to specific areas (Morse & Niehaus, 2009). Both data sets showed that suffering is prominent and that anxiety and depression predict worse mental health. Findings from both the qualitative findings and quantitative data highlight social relationships as important for mental health and, conversely, lack of social relationships as a source of suffering. The qualitative data provided many descriptions of the life situation in an NH in which many difficult experiences throughout a long life are prominent. As such, the qualitative data provided detailed information about several psychosocial aspects and experienced suffering. Additional, the supplementary component, quantitative data, showed that emotional closeness and relationships with people who share concerns and interests is important.

The combined findings call for several improvements in care among the residents, which more accurately reflects their concerns aimed at alleviating suffering.

## Discussion

This study among cognitively intact NH residents showed that the individual stories reveal that psychosocial aspects and the phenomenon of suffering are related to painful experiences during life. The quantitative data showed that symptoms of both anxiety and depression were related to mental health. The association between anxiety and depression and mental health may suggest that more symptoms of both anxiety and depression contribute to worse mental health. Other studies among NH residents (Smalbrugge et al., 2005b; Smalbrugge et al., 2006b; Rodriguez-Blazquez et al., 2012) reported associations between the presence of depression and/or anxiety symptoms and worse well-being. One explanation for our results could be that most of our respondents were widows or widowers, have multiple diagnoses and were dependent in the activities of daily living. All these circumstances may be experienced as losses, contribute to symptoms of depression (Bland, 2012) and influence mental health (Ferrell & Coyle, 2008) and the experience of suffering (Kuuppelomaki & Lauri, 1998; Ferrell & Coyle, 2008; Morse, 2011). The informants also clearly expressed the experience of suffering by telling stories containing both earlier and current life events. Morse (2011) emphasizes the significance of assisting the residents in moving from endurance to emotional release when coping with suffering. Interpersonal encounters seem to play an important role here to help the person who is suffering in this process (Yalom, 2005), and nurses must maintain a culture that includes such basic elements of nursing.

Symptoms of anxiety were related to the bodily pain subdimension of the health-related quality of life. This dimension of the SF-36 measures the intensity of bodily pain and the extent to which bodily pain interferes with normal activities (Ware, 2005). Among older NH residents, Smalbrugge et al. (2006b) found associations between anxiety and well-being. But in contrast to our study, the diagnosis of anxiety was reported and the study did not explicit focus on pain. Pain is a source of suffering (Cassel, 2004; Ferrell & Coyle, 2008) and a primary source of physical suffering (Kuuppelomaki & Lauri, 1998).

Our study indicated that higher levels of attachment and social integration are associated with higher levels of vitality and social functioning, or conversely, a lower level of attachment and social integration corresponds to lower levels of vitality and social functioning.

The positive relationship between attachment and vitality suggests that the emotional content of the relationship with significant others is an important component of vitality. In addition, the stories from the residents underline the importance of social relationships and mobility as important aspects of this. Our extended findings are in accordance with other studies that report positive associations between social support and well-being (Tseng & Wang, 2001; Bergland & Kirkevold, 2005; Drageset et al., 2009b; Rodriguez-Blazquez et al., 2012). Weiss (1973) and Weiss (1974) emphasizes that significant others are spouses and very close friends who provide the feeling of intimacy, security and peace, and a lack of significant others contributes to experiencing negative feeling as emotional loneliness. Because of the respondents’ advanced age, disability and dependence, they are more likely to have difficulty in maintaining close social contact. Social and emotional support seems to be important in combating depression in the general population (Grav et al., 2012) and loneliness among NH residents (Drageset, Kirkevold & Espehaug, 2010).

The relationship between opportunity for nurturance and emotional role limitations (whether emotional problems interfered with such social activities as visiting friends and relatives) suggests that providing more support for others would contribute to increasing role limitations. Weiss (1974) emphasizes that nurturance differs from the other provisions by enquiring whether older people themselves provide support. Responsibility for someone, usually children, gives meaning to an individual’s life in meeting obligations in various activities. One explanation for our results could be loss of the ability to give the necessary support: the relationship does not make sense to the extent that one wishes.

The individuals detailed many stories that reveal existential experiences related to suffering. These findings clearly give nuances in a holistically way and with information from the results from the quantitative supplementary component the researcher is able to build synthesis of the qualitative findings to the quantitative results which is necessary to draw conclusion. This could also clarify clinical significance and contribute to more clinically meaningful approaches. Such extended findings represent a valuable contribution in planning individual care.

### Methodological considerations

There are quantitative studies examining anxiety and depression, social support and the quality of life, but this type of study has limitations. Suffering is a life phenomenon that provides deeper understanding and different types of knowledge. Integrating quantitative and qualitative results using mixed methods could therefore provide more meaningful findings than one method alone (Tashakkori & Creswell, 2007).

Because we used a cross-sectional study design, we cannot firmly conclude on the direction of a possible causal effect or preclude that these associations are effects of other unmeasured determinants. However, the supplementary component can explain and contribute to deeper knowledge about psychosocial aspects and well-being so that this relationship appears more clearly. Further, because the study followed a cohort (*n* = 227, 30 NHs) of frail cognitively intact NH residents from 2004–2005 to 2011, the sample size was small at the end of follow-up. Despite the small sample size and low statistical power, we found meaningful statistical associations that inform the qualitative findings.

As discussed, the research problem is multifaceted and complex. The integrated research strategy offered by a mixed-methods design therefore enabled us to clarify different aspects of the phenomena (Richards & Morse, 2007). This tradition maintains that one method alone will not comprehensively answer our research questions. As such, both qualitative and quantitative methods are used to collect and analyze the data (Morse & Niehaus, 2009). In our study, the core component is qualitative data consist of details and important qualitative date about experiences related to suffering in NH. The supplementary component (the quantitative results), by clarifying the connection between anxiety and depression, informed and supported the core component (the qualitative data), as described by Morse et al. (2006). In this way, the mixed-methods design validated our findings while conforming to the rules inherent in each paradigm. Further, using a mixed-methods design with the use of a semistructured qualitative interview may increase the subjectivity of evaluation and create difficulty in replicating the study.

Two of the authors have skills primarily in the quantitative core method, and two authors were primarily qualitative researchers. In addition, a statistician was responsible for the quantitative data analysis. In this way, the researchers represented the different and overlapping research fields necessary for using a mixed-methods design. In terms of validity, we believe that our different platforms and expertise represented a critical contribution in the continuous discussion about possible interpretations during the whole research process.

## Conclusion and clinical implications

The individual stories reveal that psychosocial aspects and the phenomenon of suffering are related to painful experiences during life. Symptoms of anxiety and depression were negatively associated with mental health, and symptoms of anxiety were associated with bodily pain. Attachment and social integration were associated with vitality and social functioning. As demonstrated, the supplementary component informed and supported the core component and contributes to extending knowledge about the study topic. To improve the situation of residents, more attention should be paid to the residents’ suffering related to anxiety, depression and relationships. The challenge for health care providers is to grasp the individual experience of the patients and the meaning these experiences may have for them. This requires that health care personnel listen to each individual and help them to find their own strategies to live with the losses. By building on earlier successful strategies, patients can be helped to understand current suffering. Listening to the patients in this way requires time, patience and professional competence. A management structure that focuses on how to meet each resident’s needs individually, such as a care plan for each patient and a primary contact, is also essential. Further, it is also important that health care personnel help the individuals to participate in meaningful activities and interactions with peers, friends and family. Future studies are needed to corroborate or analyze more in depth the present results about suffering.

## Additional Information and Declarations

### Data Deposition

The following information was supplied regarding the deposition of related data: According to the Norwegian ACT 2008-06-20 no. 44: Act on medical and health research (the Health Research Act) regulated in the paragraph 7 Duty of confidentiality and 13 Main rule on consent, we cannot make the raw data available.
